# Boundaries and potentials of traditional and alternative neuroscience research methods in music therapy research

**DOI:** 10.3389/fnhum.2015.00342

**Published:** 2015-06-09

**Authors:** Andrea M. Hunt

**Affiliations:** Department of Music, Immaculata UniversityImmaculata, PA, USA

**Keywords:** music therapy, neuroscience methods, research methods, epistemology, opinion

How should music therapists engage with the enormous potential of neuroscience research? The methodological rigors usually employed in such research complicate this highly attractive arena, requiring operationalizing music and removing it from the context in which it is usually experienced (Fachner and Stegemann, 2013). Deconstructing music in this way merely addresses the neural processing of music perception and action, ignoring the holistic experience of music, which unfolds over time and is embedded in personal and situational context (Fachner, 2002). Furthermore, because music therapy by definition is an interpersonal experience involving client and therapist, and the therapy process depends “upon not merely the music, but also the client's experience of it” (p. 115, Bruscia, 2014), research methods which isolate the research subject from this interaction neglect an important component in the clinical dynamic of music therapy.

From a broader perspective, emerging research into the effects of early relationships on brain development and behavior (Schore, 2012), shows that individuals' brains have unique patterns of interacting with the world as well as perceiving and responding to the world. While cognitive neuroscience can identify some global responses to music as stimuli, the high degree of variability across individuals continues to be a serious confounding factor. In response, new research methods are exploring ways to account for individual experience in conjunction with neuroimaging (Varela, 1996) as well as how interpersonal musical interaction correlates with brain activity (Lindenberger et al., 2009).

Therefore, in this piece I will discuss researching and interpreting the behavior of the human brain in relation to music therapy contexts. I will delineate the boundaries of research methods employed in the neurosciences and discuss ways in which new, alternative methods have the potential to meaningfully elucidate clinically relevant information for music therapists.

## Cognitive neuroscience for music therapy

Cognitive Neuroscience (CNS) as a discipline has generated fascinating insights into the structure and functions of the human brain, with near-daily revelations regarding the complex nature of the nervous system. Music psychologists and CNS researchers have discovered brain structures and networks related to music processing of many kinds, including music perception, emotion and music, and sensory processing and music. Other research has focused on the effects of music training on processes such as cognition, emotion, self-regulation, learning, and neuroendocrine functions. Other discoveries include the mirror neuron system, showing how the brain processes perception and translates it into action, as well as the principle of neuroplasticity, where neural pathways can shift and become stronger with repeated use and training. All of these discoveries have had major implications in music therapy practice, especially for clinicians who work primarily with brain injury and disease. The recent media coverage (e.g., Moise, 2011) of U.S. Congresswoman Gabrielle Giffords' recovery from an attacker's gunshot wound to her left temporal lobe has highlighted ways music therapists have used this knowledge in neurological rehabilitation.

## Requirements of CNS research

CNS research in music cognition or music therapy involves the use of complex imaging systems that require stringent controls in order to obtain reliable, valid data. This involves operationalizing the stimuli—in the context of music listening studies, the music is often considered as the stimulus, while in the context of active music making, playing music is a complex neurological task. The music, and its delivery or activity, must be clearly defined, standardized, and controlled. CNS research also demands controlled designs in order to attain the best results given the limitations and strengths of the imaging methods being used. Often subjects undergo multiple (sometimes hundreds) of trials in order to obtain a large dataset, with the results averaged in order to find universal responses to that stimulus or condition. These strict protocols are necessary to answer specific research questions, with little room for individualizing approaches to fit a clinical music therapy situation.

Furthermore, subjects' movements may be restricted, or they cannot use particular materials or musical instruments while undergoing an imaging study, due to the nature and constraints of the imaging equipment. For example, the magnetic field generated by an fMRI machine would preclude investigating the subject's playing of any instruments that contain metal. Despite these limitations, researchers interested in active music making have found ways to work around these limitations, by using materials safe for the scanner (e.g., a non-ferromagnetic piano keyboard for use in fMRI) or having subjects play instruments that are not only compatible with the imaging method, but also do not require much head movement that would result in artifacts (e.g., saxophonists playing during EEG acquisition in Babiloni et al., 2011). Each imaging method also has its strengths and limitations in its temporal and spatial resolution; thus researchers should choose the most appropriate imaging method for the research question, given the kind of data the imaging can obtain.

## Integrating music therapy and CNS

Research in recent years has demonstrated ways that music therapy and neuroimaging can work well together, particularly for rehabilitation from neurologic injury. For example, Altenmüller et al. (2009) used EEG to show how music therapy can improve cortical connections and activity in stroke patients. Schlaug et al. (2009) used diffusion tensor imaging to reveal neurological changes after melodic intonation therapy for persons with left-hemisphere stroke damage. These highlights are in addition to more comprehensive reviews of music therapy in rehabilitation (Hurt-Thaut, 2009; Leins et al., 2009). More recently, researchers have used neuroimaging to discover lasting changes in brain functioning after 18 sessions of music therapy for depression (Fachner et al., 2013) and to identify brain responses to different types of music intervention for pain (Hauck et al., 2013).

## Limits of CNS for music therapy

While these studies are encouraging, readers must note that CNS methods have limitations, many of which have been summarized in Christensen (2012). Primarily, operationalized music “stimuli” and the resultant designs often lack ecological validity. Many studies utilize synthesized music or tones, or short segments of music. It is rare for studies to use complete pieces of music. Furthermore, imaging equipment restricts or does not work well with body movement, limiting naturalistic ways that subjects may move while listening to or playing music. Also, because equipment is expensive and specialized, it is often located in a medical setting or laboratory, a context far removed from where clients would usually encounter music therapy. In addition, many CNS studies do not adequately report the sources of the music selections used in the research, making it difficult to interpret findings.

Aside from these methodological restrictions, the epistemological assumptions of CNS research are also restricted. Researchers assume there are universal responses to the music conditions, and dismiss outlier responses as statistical noise. There is no room for investigating unique brain responses to the experimental conditions. Music is assumed to be an object which can be operationalized as a “stimulus,” this ignores the socially-constructed meanings of music which are created within and across cultures and groups. When researchers ignore these meanings in their designs and simply create segments of tonal or rhythm patterns as their stimuli, they are really examining the brain's responses to tonal and rhythm patterns–not music.

The socially-constructed nature of music is directly related to the field of music therapy, because music therapy involves a relationship between client and therapist (Bruscia, 2014). This relationship involves the intersubjective nature of music, along with nonverbal communication and social and environmental context. These are all significant factors in the music therapy experience which must be included in order to assure ecological and sociological validity. Translating CNS research to the practice of music therapy therefore requires that the client-therapist relationship be taken into account in the research question, design, and interpretation of results.

## The future for music therapy research

For CNS to incorporate the interpersonal, subjective, and contextual factors inherent in music therapy, researchers must first be very clear about their epistemological stances in their research, while also considering other perspectives. Each perspective has strengths and limitations, and requires appropriate expertise in a research context.

**Figure 1:**
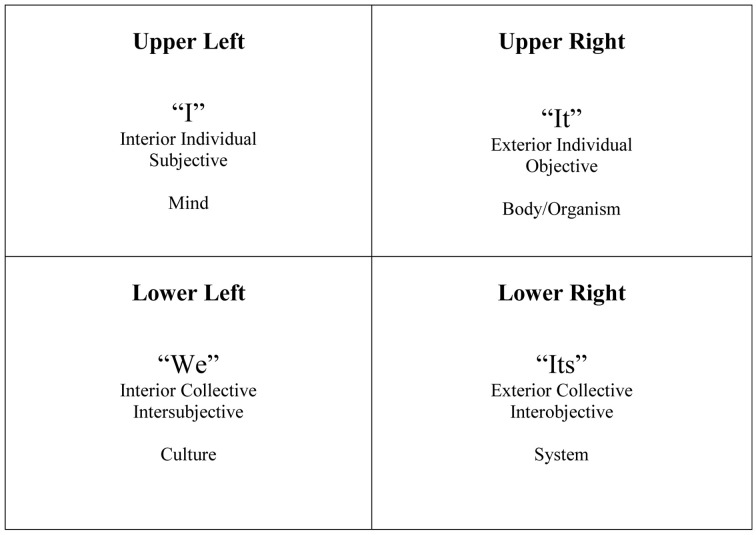
**Wilber's four quardrants**.

The philosopher Wilber (2000) has created a model to help conceptualize phenomena in all its forms and permutations (Figure 1). This model, called the Four Quadrants, has been applied to music therapy as well (Bruscia, 1998) to help delineate different clinical phenomena and approaches. The quadrants are organized in a matrix of “Interior” vs. “Exterior” phenomena, combined with “Individual” vs. “Collective” phenomena. For this paper, I will focus on the top two quadrants, “Exterior-Individual” and “Interior-Individual.”

Traditional scientific research, including experimental designs and any approach which involves the collection and analysis of observable, measurable data from individuals, is located in the Upper Right quadrant (the Exterior-Individual/“It” region). This is also where traditional CNS research is located—music and related behaviors are viewed as objects, and brain responses to it are objectively measured and analyzed. Music therapy approaches located in this quadrant include behavioral interventions and any intervention that focuses on observable, measurable outcomes.

The Upper Left quadrant (Interior/Individual/“I” region) contains phenomena including the individual's subjective feelings, experiences, memories, and values. Research located in this quadrant includes phenomenology and heuristic research, while the music therapy methods here include psychoanalytic or humanistic approaches that emphasize the individual's internal experience which typically cannot be measured or observed objectively.

The future of music therapy research needs to address several kinds of phenomena. First, it needs to account for the variability among subjects' neurological responses. For example, research should consider cultural and personal context in neurological development, which can lead to unique patterns of perception and response (Schore, 2012). Second, research needs to account for human interaction in the music therapy experience—that is, understanding “music” as a verb rather than a noun/stimulus (Small, 1998). In particular, research should attempt to address client-therapist interaction during clinical experiences. In other words, the future of music therapy research in the neurosciences should involve perspectives from quadrants other than the Upper Right.

Some research methods from these quadrants have already been developed in the neurosciences. One such method is neurophenomenology (Varela, 1996). This approach originated as a biological investigation of subjectivity and consciousness, but evolved to include other phenomena including an integrated investigation into the biological and subjective experience of a guided imagery and music session (Hunt, 2011). The approach integrates objective data and subjective experience in individuals, holding that (1) the first-person experience is irreducible, (2) the first-person investigation must be rigorous, and (3) the first- and third-person perspectives are equally important. In the Hunt (2011) study, the researcher collected phenomenological data from each participant's imagery report of the music therapy session, and correlated it with EEG coherence data to generate integrated descriptions of biological and subjective experience during the sessions.

Hyperscanning is another research method with great potential for music therapy. Here, imaging data are collected simultaneously from two or more subjects and analyses focus on ways that the brain data synchronize with each other around shared experiences or events. For example, Sänger et al. (2012) examined the EEGs of two musicians playing guitar duets and found that phase locking indices were high when the musicians were setting the initial tempo, as well as immediately prior to, and after onset of, playing. More recent studies have utilized hyperscanning with functional near-infrared spectroscopy (fNIRS; see review in Scholkmann et al., 2013) with great success. Integrating multiple participants' data could even be used within a neurophenomenological approach in order to understand what participants undergo while making music together in music therapy.

In addition to these new methodological and paradigmatic approaches, portable EEG (Wascher et al., 2013; DeVos and Debener, 2014) and wireless fNIRS (Scholkmann et al., 2013) can permit *in situ* neuroimaging, thereby increasing ecological validity. While these imaging options have limitations in terms of spatial resolution and standardized norms for comparison, they are relatively inexpensive compared to fMRI, PET, and SPECT technology. Research designs and questions which focus on these portable devices' imaging strengths could lead to greatly increased understanding of neurological activity during music therapy experiences.

With the advent of both innovative neuroimaging technology and new research perspectives and designs, music therapists are uniquely poised to undertake ground-breaking research into ways that music therapy affects and benefits clients. However, prevailing thinking around CNS research could divert attention away from these possibilities, and instead focus research on Upper Right phenomena alone. While this research undoubtedly has been beneficial, it has limited translatability to music therapy experience and practice. Let us not limit our understanding to one perspective; instead let us step into new perspectives, as we do with our clients, willingly looking at the world from a new place, with new eyes, and new comprehension.

### Conflict of interest statement

The author declares that the research was conducted in the absence of any commercial or financial relationships that could be construed as a potential conflict of interest.
